# Cost-effectiveness analysis of a randomized study of depression treatment options in primary care suggests stepped-care treatment may have economic benefits

**DOI:** 10.1186/s12888-019-2223-3

**Published:** 2019-08-05

**Authors:** Charles Yan, Katherine Rittenbach, Sepideh Souri, Peter H. Silverstone

**Affiliations:** 1 Institute of Health Economics, 1200 – 10405 Jasper Avenue, Edmonton, Alberta T5J 3N4 Canada; 2 Department of Psychiatry, Addiction & Mental Health Strategic Clinical Network, Alberta Health Services, University of Alberta, 10030 107 St, NW, Edmonton, Alberta T5J 3E4 Canada; 3 Cumming School of Medicine, University of Calgary, 3330 Hospital Drive NW, Calgary, Alberta T2N 4N1 Canada; 4 Department of Psychiatry, University of Alberta, 8440 112 St NW, Edmonton, Alberta T6G 2B7 Canada

**Keywords:** Cost-effectiveness, Mental illness, Depression, Primary care, Quality of life

## Abstract

**Background:**

The stepped-care pathway (SCP) model has previously been found to be clinically effective for depressive disorder in some studies, but not all. Several groups have suggested that a stepped-care approach is the most appropriate in primary care. There is relatively little information, however, regarding which specific stepped-care pathway may be best. This analysis aimed to determine cost-effectiveness of a stepped-care pathway for depression in adults in primary care versus standard care (SC), treatment-as-usual (TAU), and online cognitive behavioural therapy (CBT).

**Methods:**

We conducted a randomized trial with 1400 participants and 12-week follow-up to assess the impact of the four treatment options on health-related quality of life and depression severity. Costs for the groups were calculated on the basis of physician, outpatient, and inpatient services using administrative data. We then calculated the incremental cost-effectiveness ratios using this information. Cost-effectiveness acceptability curves and incremental cost-effectiveness scatterplots were created using Monte Carlo simulation with 10,000 replications. A subgroup analysis was conducted for participants who screened as depressed at baseline.

**Results:**

For all participants, TAU was the most expensive followed by CBT, SC, and SCP. QALYs were highest in SCP, followed by SC, CBT, and TAU. In the depressed subgroup, TAU was still the most expensive, followed by SC, SCP, and CBT, while QALYs were still highest in SCP, followed by SC, CBT, and TAU. The cost-effectiveness acceptability curves suggested that SCP had a higher probability for cost-effectiveness than the other three alternatives in all participants. In the depressed subgroup, CBT was associated with the highest probability of cost-effectiveness for a willingness-to-pay cut-off of less than approximately $50,000, while SCP was the highest at a cut-off higher than $50,000. There is considerable uncertainty around the cost-effectiveness estimates.

**Conclusions:**

Our analysis showed that even where there are no clinically significant differences in health outcomes between treatment approaches, there may be economic benefit from implementing the stepped-care model. While more work is required to identify the most clinically effective versions of a stepped-care pathway, our findings suggest that the care pathway may have potential to improve health care system value.

**Trial registration:**

NCT01975207. The trial was prospectively registered on 4 November 2013.

**Electronic supplementary material:**

The online version of this article (10.1186/s12888-019-2223-3) contains supplementary material, which is available to authorized users.

## Background

Depressive disorder has one of the highest degrees of disease burden in established market economies such as Canada [1–4]. This is due to such factors as incidence (between 10 and 20% of patients who attend their primary care physicians) [5] and effects such as high rates of unemployment and disability in individuals with major depressive disorder in Canada [6]. Reduction of the considerable disease burden, and its associated economic costs, is a key objective for depression management.

A potential approach to more effective depression management is to universally screen all adults attending primary care and communicate the screening results to the clinic staff for appropriate interventions and/or treatments [7, 8]. When depression has been identified in primary care practice, standard care (SC) can involve a combination of ‘watchful waiting’, advice, psychotherapy, medication, or referral to a specialist [9, 10]. Of the psychological treatments available, cognitive behavioural therapy (CBT) is the most commonly used, both in person and electronically via computer [11, 12]. Given the variety of treatment options, for many years several groups have suggested that a stepped-care treatment approach is the most appropriate in primary care [13, 14]. The stepped-care pathway (SCP) model usually includes both antidepressant medication and psychosocial interventions, and has previously been found to be clinically effective for depressive disorder in some studies [15–17], but not all [14, 18]. Furthermore, the implementation of the stepped-care treatment can be both resource and staff intensive, and the specific components of individual programs vary widely [19, 20].

There is also relatively little information regarding which specific stepped-care pathway may be best [21]. We have previously reported clinical findings from a randomized, controlled trial (RCT) in adults attending two primary clinics, in which over 1400 patients were screened for depression and randomized into one of four groups [20]: (1) a standard care (SC) control group in which neither patient nor physician knew the results of depression screening; (2) a treatment-as-usual (TAU) group, where physicians were made aware of depression scores, but no guidance regarding treatment was given; (3) an online CBT group in which patients who were depressed were given login information for a well-tested online CBT program; and (4) a stepped-care pathway (SCP). The exception to this randomization was that any participant who scored as high risk for self-harm at any screening time was referred directly to their primary care provider. The stepped-care program we used was developed in Calgary, Alberta, Canada, and was based on existing literature. This program had previously been utilized with 158 patients in an open-label study in five primary care locations during the period 2010–2011 [20, 22].

In our previously reported study [20], the primary clinical outcome was the changes in depression scores, measured by the Patient Health Questionnaire (PHQ-9) [23–25]. The PHQ-9 is a nine-question instrument commonly used for measuring the severity of depression in a primary care setting. Patients’ responses to the questions are converted into scores, ranging from 0 to 27 with 0 representing minimal disorder and 27 being the most severe. Patients with a PHQ-9 score greater than 10 are deemed to have moderate to severe depression. Health-related quality of life (HRQoL) was also measured using the EuroQol-5-dimension with a five-level scale (EQ-5D-5 L) [26–28]. All patients were measured at baseline and 12-week post-randomisation, while patients who were depressed at baseline had an additional measurement at six weeks [20]. Interestingly, our results showed no differences in clinical outcomes between any of the four groups [20]. However, we were aware that no previous RCT had examined possible cost implications of the various treatment options, including stepped-care, and had therefore sought to obtain this data a priori, with appropriate ethics committee and subject approval. Therefore, the aim of the current publication is to report on the estimated medical costs of adults who visited their primary care physician and were screened for depression, and to determine whether the stepped-care treatment was cost-effective compared to other approaches both for those who screened positive for depression symptoms and the entire population included in the study.

## Method

### Study design and participants

In this randomised, controlled trial [20], we recruited participants from two primary care clinics in Alberta, Canada. Study groups were block randomized by day (days were randomly assigned to one of the four arms using a random number generator). We assessed participants aged 18 and above, who were able to provide informed consent. The Health Research Ethics Review Board at the University of Alberta approved the study protocol on 30th July 2013. This trial was registered with Clinical Trials database, https://clinicaltrials.gov/ct2/show/NCT01975207 Identifier: NCT01975207. Details on methods employed in the trial have been published previously [20] and for that reason are only summarized here.

### Study arms

Participants were assigned to one of four arms. In Arm 1, standard care (SC), the results of PHQ-9 screening were not communicated to the patient or their physician, unless they scored positive for a risk of self-harm. In Arm 2, treatment as usual (TAU), participants who scored greater than 10 on the PHQ-9 had their scores communicated to them and to their physicians for follow up without recommendations from the study team. Arm 3 was TAU plus online cognitive behavioural therapy (iCBT). In this arm, participants who scored greater than 10 on the PHQ-9 had their scores communicated to them and to their physicians for follow-up, and patients were provided login information for a free internet-based CBT [29] and encouraged to use it. Participants who scored greater than 10 on the PHQ-9 in Arm 4, known as stepped-care pathway (SCP), had their scores communicated to them and their physicians and were offered a pre-determined treatment as indicated by the depression stepped-care pathway, described in detail elsewhere [20]. Participants in all arms had information collected on their health-related quality of life and symptoms of depression at baseline and week 12 using the EuroQol-5 dimension (EQ-5D-5 L) and the PHQ-9 measure. Those who scored over 10 on the PHQ-9 at baseline also completed the data collection at six weeks, and if at any point any participant scored “at risk” for self-harm, they were referred for urgent treatment.

### Data collection

Patients completed the self-reported Patient Health Questionnaire-9 (PHQ-9), an instrument that can be used to assist in screening and monitoring the severity of depressive symptoms [23], as well as the EQ-5D-5 L at baseline and 12 weeks post-randomization. The EQ-5D-5 L is a standardized measure of health status developed by the EuroQol Group that provides respondents with a descriptive system to classify their health status based on five dimensions: mobility, self-care, usual activities, pain/discomfort, and anxiety/depression [27, 30]. This measurement provides a utility score for each of the EQ-5D-5 L health states between values of 0 and 1, with 1 representing the best (perfect) health state and 0 the worst (death) state. The analysis was performed from the perspective of health care payer, and the costs and resource utilization included in the analysis were for physician, outpatient, and inpatient services. Physician services include all activities performed in primary, outpatient, and inpatient care settings. Inpatient and outpatient costs cover all activities other than physician service in these settings. Examples of these costs include salaries, drugs, medical and surgical supplies, administration, and support services. We collected data during the periods of 12 weeks pre-randomization, 12 weeks post-randomization, and from 12 weeks to 1-year post-randomization. While some data were also collected in depressed patients at week six (approximately 20% of the total sample), it is not included in the present analysis. Data on health care system usage were retrieved from health administrative databases that provide individual patient information such as age, gender, cost, diagnostics, and service date. Since there is a single health care service in the province of Alberta, this information comprised all relevant health spending for the study patients with the exception of private psychological services, which we are unable to include in the analysis. In other words, the analysis included direct medical costs only, while other societal costs, such as productivity losses and transportation, were not included.

### Statistical analysis

The analysis followed the guidelines set by the International Society for Pharmacoeconomics and Outcomes Research (ISPOR) Good Research Practices Task Force [31, 32]. An intention-to-treat (ITT) approach that considered all participants allocated to each intervention arm was applied for the analysis; this approach was utilized to avoid bias [31, 33]. Missing data were handled using multiple imputation, which reflects inherent uncertainty when replacing missing data [31, 32, 34]. The data from each of the four intervention arms were further separated into a ‘depression’ subgroup of participants, who scored greater than 10 on the PHQ-9 at baseline. The analysis focuses on the depression subgroup as this is the group that may have received clinical benefit from the treatment options, since those who did not have symptoms of depression had no need for the interventions. An overall PHQ-9/EQ-5D-5 L score was estimated for each intervention arm. Imbalances in baseline PHQ-9 and EQ-5D-5 L scores were accounted for using ordinary least squares (OLS) regression in estimating their values at 12-week post-randomization. The difference between intervention arms was tested using a one-way ANOVA test, the difference between each pair of intervention arms was tested using a pairwise comparison of mean, and the difference between baseline and 12-week post-randomisation for each intervention arm was tested using paired t-test for these scores [35].

Data investigation indicated unusually higher costs for some participants, known as outliers, compared with the average cost for each study group. Outliers skew average costs and are unlikely to represent the true average expenditure for healthcare services [36, 37]. The outliers in each period and intervention arm were trimmed. We used a traditional univariate boxplot to trim physician and outpatient costs, with cost data beyond 1.5 times the interquartile range (IQR) being excluded. We did not trim outliers for inpatient costs because of the limited available data, as there were only a few participants admitted to hospital. A generalized linear model (GLM) was applied to adjust for imbalance in baseline characteristics such as age, gender, and PHQ-9 scores in estimating costs at 12-week and one-year post-randomization. The GLM extends the linear modelling approach to data that are not normally distributed. We used a gamma distribution as the family suitable when fitting skewed healthcare cost data [38]. The link function used in GLM specifies how the mean of the dependent variable depends on the predictors. In the analysis we used identity link function, implying a linear relation between the cost and predictors. Predicted cost values from the GLM were used to represent the costs for each period and each intervention arm. Discounting was not required, as the study time horizon was just one year. All costs were adjusted to a standard price year of 2017, using the Alberta Consumer Price Index (CPI).

We compared the costs and cost-effectiveness of the intervention arms at one year to capture the economic effect of the alternatives. We used quality-adjusted life-years (QALYs) as the primary outcome to measure health effectiveness over 12 months. We calculated QALYs as the area under the curve defined by the EQ-5D-5 L utility scores at baseline and one year. The RCT did not collect the EQ-5D-5 L data after 12-week post randomisation, and we therefore assumed the utility scores over the time point remained unchanged. This assumption implies that treatment effect is maximized in the first 12 weeks. We conducted a sensitivity analysis on two different scenarios to test the impact of this assumption.

Health care utilization data for participants in the RCT indicated that, during the year after randomization, a participant received either no medical services or a combination of physician, outpatient, and/or inpatient services. As shown in Table 1, approximately 1 to 2% of participants did not receive any medical services; 40 to 45% had physician visits only; 40 to 48% had a combination of physician visits plus outpatient visits; 0 to 1.7% had a combination of physician plus inpatient admission; and 9 to 17% received medical services from all the sectors (physician visits, outpatient visits, and inpatient admission).

**Table 1:** Number of participants receiving physician, outpatient, and/or inpatient services during first year after randomization

Arm	No services	Physician only	Physician + outpatient	Physician + inpatient	Physician + outpatient + inpatient	Total
All participants						
SC	4 (1.0%)	179 (43.4%)	176 (41.99%)	3 (0.73%)	53 (12.9%)	412
TAU	8 (2.0%)	160 (40.3%)	160 (39.80%)	2 (0.5%)	69 (17.4%)	397
ICBT	4 (1.0%)	188 (45.3%)	164 (37.83%)	7 (1.69%)	59 (14.2%)	415
SCP	3 (1.6%)	75 (41.0%)	88 (48.08%)	na	17 (9.3%)	183
All	19 (1.4%)	602 (42.8%)	588 (40.94%)	12 (0.85%)	198 (14.1%)	1407
Depressed subgroup						
SC	0 (0%)	21 (37.5%)	28 (50%)	na	7 (12.5%)	56
TAU	2 (3%)	21 31.8(%)	35 (53%)	na	8 (12.1%)	66
ICBT	0 (0%)	22 (44%)	23 (46%)	na	5 (10%)	50
SCP	0 (0%)	12 (35.3%)	18 (52.9%)	na	4 (11.8%)	34
All	2 (1%)	76 (36.9%)	104 (50.5%)	na	24 (11.7%)	206

The SCP group had a small likelihood of using expensive inpatient services in this study, which significantly impacted overall spending observed during the trial. In order to verify that the results of this analysis were not biased due to this decrease in inpatient spending, we developed a decision tree model to capture the cost of each combination of services for participants. This model splits participants into one of the five combinations reported in Table 1 and assigns the probabilities of service use to each group. We used the decision tree in the base case analysis, and because the likelihood of inpatient use is the main component driving difference in cost-effectiveness, we conducted a one-way sensitivity analysis to assess the impact of changing the likelihood.

The incremental cost-effectiveness ratio (ICER) was calculated as the ratio of differences in mean costs and mean number of QALYs [39]. The differences in means were assessed using Monte Carlo Simulation, a mathematical technique to address the uncertainty of the cost effectiveness. The input cost and effectiveness data were repeatedly modelled for 10,000 iterations on the basis of known probability distributions for generating the outcomes [31, 40–42]. Based on results from the Monte Carlo simulations, we constructed the cost-effectiveness acceptability curves and incremental cost-effectiveness scatterplots to help the understanding of the uncertainty of the ICER [39, 43]. In addition to the analysis performed on the overall participants, the cost-effectiveness analysis was performed in a subgroup of participants who scored greater than 10 on the PHQ-9 at baseline [31, 44]. The statistical analysis was performed with the Stata software package (Release 13.1) for Windows, and decision analytic modelling analysis was performed with TreeAge Pro 2015 software (TreeAge Software, Inc).

## Results

We recruited 1400 participants between November 2013 to December 2014, randomly allocating 412 (29%) to SC, 397 (28%) to TAU, 415 (29%) to ICBT, and 183 (17%) to SCP. Note that the numbers in each arm were not balanced, as not all clinics were able to offer the stepped-care treatment. Of the 1400 total subjects, 206 participants scored greater than 10 on the PHQ-9 at baseline (referred to as “depressed” henceforth), with 56 (27%), 66 (32%), 50 (24%), and 34 (17%) being depressed in Arms 1 to 4, respectively. Table 2 presents baseline demographics and outcomes measured in terms of PHQ-9 and EQ-5D-5 L. The mean (s.d.) age was 47 (17) years in all participants and 45 (15) years in depressed ones, and the majority was female (73% in all participants and 74% in depressed ones). More data are available in our Additional file 1.

**Table 2:** Description of the sample

All participants					
	SC (*n* = 412)^§^	TAU (*n* = 397)	ICBT (*n* = 415)	SCP (*n* = 183)	All (*n* = 1407)
Age (s.d.)	46.9 (17.1)	45.5 (16.3)	46.6 (16.8)	52.3 (17.6)	47.1 (17.0)
Female (%)	75%	78%	70%	61%	73%
PHQ-9 (s.d.), baseline	4.55 (4.95)	4.98 (5.19)	4.2 (4.61)	4.86 (5.54)	4.61 (5.01)
PHQ-9 (s.d.), 12-week	3.85 (2.94)	4.12 (3.09)	3.65 (2.74)	4.04 (3.29)	3.89 (2.98)
within group difference (95% CI)*	0.69 (0.50 to 0.98)	0.88 (0.67 to 1.08)	0.55 (0.37 to 0.73)	0.82 (0.49 to 1.15)	0.72 (0.61 to 0.82)
EQ-5D-5 L (s.d.), baseline	0.86 (0.11)	0.84 (0.14)	0.86 (0.12)	0.86 (0.13)	0.86 (0.12)
EQ-5D-5 L (s.d.), 12-week	0.88 (0.06)	0.87 (0.08)	0.88 (0.07)	0.88 (0.08)	0.88 (0.07)
within group difference (95% CI)**	0.022 (0.017 to 0.026)	0.03 (0.024 to 0.035)	0.022 (0.017 to 0.027)	0.022 (0.014 to 0.03)	0.024 (0.021 to 0.027)
Depressed participants (PHQ-9 > 10 at baseline)					
	SC (*n* = 56)	TAU (*n* = 66)	ICBT (*n* = 50)	SCP (*n* = 34)	All (*n* = 206)
Age (s.d.)	44.7 (16.3)	44.8 (14.2)	42.2 (13.8)	49.0 (16.4)	44.8 (15.1)
Female (%)	80%	70%	70%	74%	74%
PHQ-9 (s.d.), baseline	14.73 (4.17)	14.83 (3.91)	14.28 (3.76)	14.85 (3.84)	14.67 (3.91)
PHQ-9 (s.d.), 12-week	9.9 (2.48)	9.94 (2.31)	9.64 (2.24)	9.98 (2.28)	9.86 (2.32)
within group difference (95% CI)*	4.83 (4.37 to 5.28)	4.85 (4.46 to 5.24)	4.64 (4.21 to 5.08)	4.88 (4.33 to 5.42)	4.8 (4.58 to 5.02)
EQ-5D-5 L (s.d.), baseline	0.68 (0.18)	0.63 (0.2)	0.67 (0.22)	0.68 (0.21)	0.66 (0.2)
EQ-5D-5 L (s.d.), 12-week	0.78 (0.1)	0.74 (0.12)	0.77 (0.13)	0.78 (0.12)	0.76 (0.12)
within group difference (95% CI)**	0.095 (0.075 to 0.115)	0.118 (0.096 to 0.139)	0.1 (0.075 to 0.126)	0.094 (0.064 to 0.125)	0.103 (0.092 to 0.115)

§: This table represents numbers of patients at baseline. Patient numbers at 12-week are available in the Additional file 1

*: There was significant improvement between baseline and 12-week post-randomization (*p* = 0.015 for SC, 0.004 for TAU, 0.037 for ICBT, and 0.086 for SCP in all participants and < 0.001 in depressed participants). There was no statistically significant difference between groups at baseline and 12 weeks (*p* > 0.1 in all participants including depressed participants)

**: There was significant improvement in EQ-5D-5 L scores between baseline and 12-week post-randomization (*p* < 0.001 for SC, < 0.001 for TAU, 0.002 for ICBT, and 0.066 for SCP in all participants and *p* < 0.002 for SC, < 0.001 for TAU, < 0.007 for ICBT, and 0.033 for SCP in depressed participants). All pairwise differences of the means among the intervention arms were not statistically significant (*p* > 0.5)

We found statistically significant improvement in PHQ-9 and EQ-5D-5 L from baseline to 12-week post randomisation in all arms (Table 2). In all participants, the mean change between baseline and 12-week was 0.72 (95% CI 0.61–0.82) in PHQ-9 and 0.024 (95% CI 0.021–0.027) in EQ-5D-5 L. As expected, the amount of improvement was greater in depressed participants, for whom the mean change was 4.8 (95% CI 4.58–5.02) in PHQ-9 and 0.103 (95% CI 0.092–0.115) in EQ-5D-5 L. There was no evidence of superiority in clinical effectiveness between arms.

For the economic analyses, we used the decision tree model to estimate the cost for inpatient, outpatient, and physician services for all participants and the subgroup of depressed participants, during the one-year period post-randomization (Table 3). The costs in participants who screened as depressed at baseline were higher compared with all participants over the year. We also compared inpatient, outpatient, and physician costs for depressed and non-depressed patients over three periods: 12 weeks pre-randomization (prior to screening), 12 weeks post-randomization (treatment duration), and 12 weeks to one-year post-randomization (Table 4). We found evidence of difference in costs for all the service sectors and over all the time periods with one exception. That one exception is inpatient cost from 12 weeks to one-year, where the cost difference was not statistically significant. As mentioned, few participants were admitted to inpatient care during the study.

**Table 3:** Costs for physician, outpatient, and inpatient services for one year after randomization

Study arm	Overall		Depressed subgroup	
	*n*	mean (s.d.) [range]	*n*	mean (s.d.) [range]
Inpatient Stay				
SC	56	8472.98 (4916) [2242 to 23,468]	7	11,953.99 (4257) [7091 to 16,843]
TAU	70	8158.80 (5819) [1132 to 32,349]	7	14,771.99 (6465) [5837 to 27,309]
ICBT	66	8477.81 (5340) [2119 to 33,459]	5	9357.69 (2698) [6232 to 13,415]
SCP	17	10,670.27 (3994) [4060 to 16,724]	4	10,820.99 (2660) [8528 to 14,574]
All	209	8548.00 (5309) [1132 to 33,459]	23	12,050.19 (4787) [5837 to 27,309]
Outpatient				
SC	198	1036.58 (386) [535 to 2123]	33	1256.53 (498) [593 to 2123]
TAU	200	1002.32 (392) [497 to 2127]	38	1188.6 (428) [637 to 2127]
ICBT	193	945.89 (340) [480 to 1711]	24	1101.8 (420) [488 to 1711]
SCP	90	1144.49 (611) [103 to 2993]	21	1800.39 (648) [641 to 2993]
All	681	1015.07 (416) [103 to 2993]	116	1300.72 (543) [488 to 2993]
Physician				
SC	395	683.75 (241) [89 to 1300]	52	802.56 (285) [200 to 1300]
TAU	375	758.17 (269) [250 to 1534]	60	996.41 (326) [341 to 1534]
ICBT	394	667.49 (214) [160 to 1045]	48	787.08 (216) [160 to 1045]
SCP	172	559 (207) [173 to 901]	31	633.6 (648) [234 to 901]
All	1336	683.78 (245) [89 to 1534]	191	832.14 (298) [160 to 1534]

**Table 4:** Inpatients, outpatient and physician costs for depressed and non-depressed patients^§^

Period	Depressed patients		Non-depressed patients	
	n	Mean (s.d.) [Range]	n	Mean (s.d.) [Range]
Inpatient				
12-Week Prior**	10	10,010.70 (2812) [5720 to 13,469]	51	8517.04 (3913) [3338 to 16,933]
12-Week Post*	7	9544.90 (2272) [6216 to 11,890]	76	5664.30 (2229) [2768 to 13,550]
One year**	17	9441.57 (3339) [5025 to 15,311]	116	9429.63 (4577) [2968 to 19,157]
Outpatient				
12-Week Prior*	57	727.89 (195) [141 to 1057]	245	651.66 (105) [147 to 774]
12-Week Post*	67	732.77 (136) [488 to 1055]	265	571.43 (66) [355 to 765]
One year*	91	1118.55 (242) [641 to 1938]	441	881.98 (156) [103 to 1371]
Physician				
12-Week Prior*	147	282.32 (52) [110 to 401]	734	228.04 (35) [56 to 322]
12-Week Post*	178	346.92 (73) [151 to 510]	997	260.22 (44) [89 to 374]
One year*	157	619.02 (149) [251 to 1024]	964	513.65 (89) [213 to 781]

§: Depressed and non-depressed patients were those who scored greater than 10 and less than 10 on PHQ-9 at baseline, respectively

*: One-way ANOVA test: statistically significant difference between depressed and non-depressed patients (*p* < 0.001)

**: One-way ANOVA test: no statistically significant difference between depressed and non-depressed patients (*p* > 0.25)

*s.d*. Standard Deviation, Range: Minimum to Maximum

**Fig. 1:**
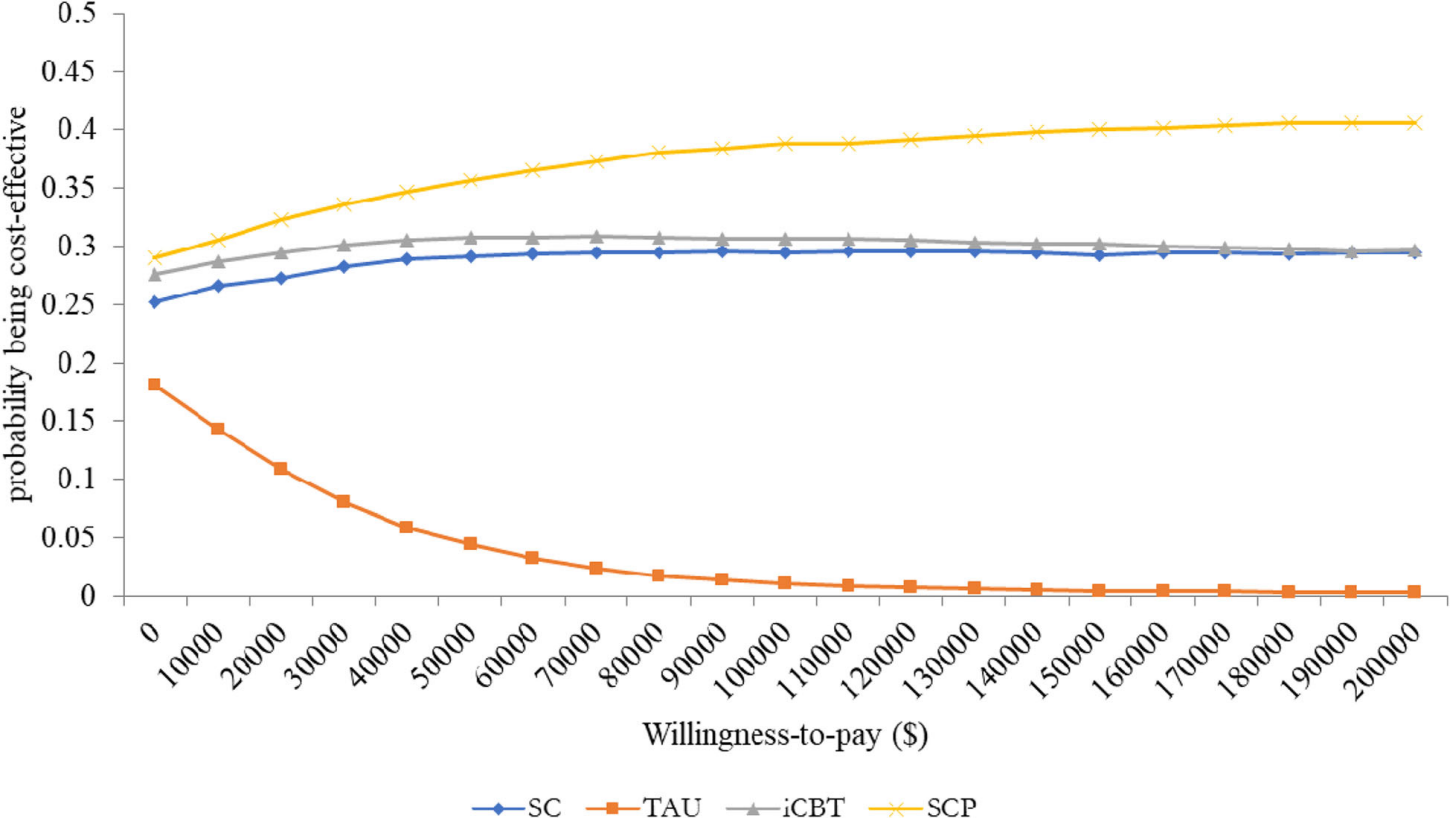
Cost-effectiveness acceptability curves for all participants

**Fig. 2:**
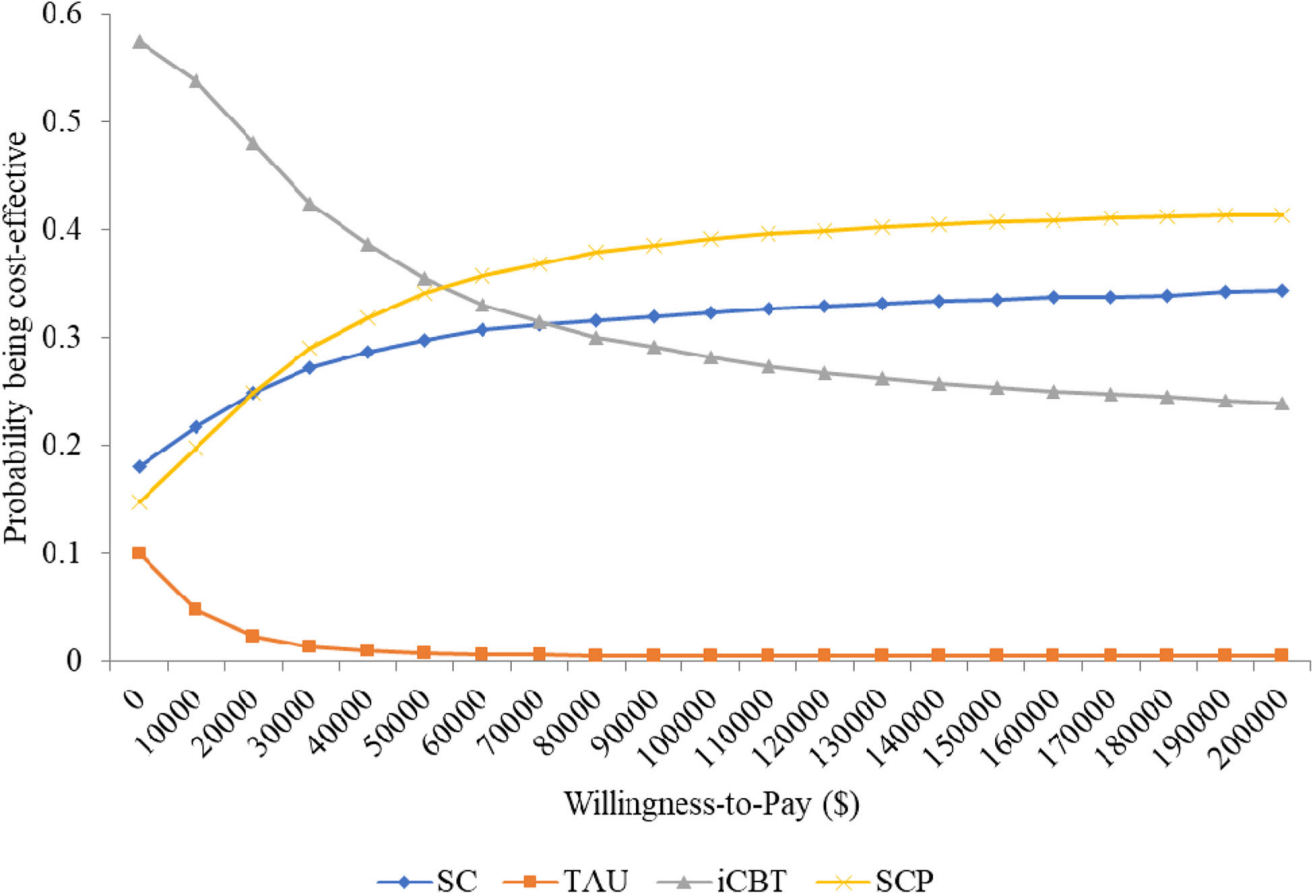
Cost-effectiveness acceptability curves for depressed participants

The results of the cost-effectiveness analysis (Table 5) show that for all participants, TAU was associated with the highest total cost, followed by iCBT, SC, and SCP with SCP being the least costly alternative. Further, QALY was highest in SCP, followed by SC, iCBT, and TAU. In those who screened as depressed at baseline, TAU was still the most expensive, followed by SC, SCP, and iCBT with iCBT being the least costly alternative rather than SCP. However, QALY was still highest in SCP, followed by SC, iCBT, and TAU. The cost-effectiveness acceptability curves revealed that, for the entire cohort, SCP was associated with the highest probability of being cost-effective over a range of willingness-to-pay from 0 to $200,000 (Fig. 1). In the subgroup of depressed participants, ICBT was associated with the highest probability of cost-effectiveness for a willingness-to-pay from 0 to approximately $50,000, while SCP was highest at a willingness-to-pay greater than $50,000 (Fig. 2).

**Table 5:** Mean cost and QALY per participant

Sector	SC	TAU	ICBT	SCP
All participants				
Physician Mean (s.d.)	$678 (239)	$744 (263)	$661 (213)	$548 (205)
Outpatient Mean (s.d.)	$572 (212)	$575 (228)	$490 (177)	$653 (351)
Inpatient Mean (s.d.)	$1096 (630)	$1421 (1006)	$1202 (758)	$990 (372)
Total (s.d.)	$2346 (655)	$2740 (968)	$2353 (767)	$2191 (539)
Cost Difference (95% CI) (SCP vs. others)	-$155 (−173-- -138)	-$549 (− 585-- -514)	-$161 (− 200 -- -123)	–
QALY (s.d.)	0.881 (0.003)	0.870 (0.004)	0.881 (0.003)	0.882 (0.005)
QALY Difference (95% CI) (SCP vs. others)	0.0001 (0 -- 0.0004)	0.0124 (0.012 -- 0.013)	0.0003 (0 -- 0.0008)	–
Depressed participants				
Physician Mean (s.d.)	$794 (281)	$966 (316)	$784 (217)	$634 (210)
Outpatient Mean (s.d.)	$787 (314)	$770 (278)	$618 (234)	$1163 (416)
Inpatient Mean (s.d.)	$1497 (535)	$1779 (780)	$1119 (321)	$1279 (315)
Total (s.d.)	$3078 (677)	$3516 (890)	$2522 (455)	$3075 (565)
Cost Difference (95% CI) (SCP vs. others))	-$3 (−20—14)	-$440 (−472-- -409)	$554 (525--582)	–
QALY (s.d.)	0.766 (0.014)	0.730 (0.014)	0.758 (0.017)	0.767 (0.021)
QALY Difference (95% CI) (SCP vs. others)	0.0013 (0--0.0025)	0.037 (0.035--0.039)	0.0097 (0.008--0.012)	–

**Fig. 3:**
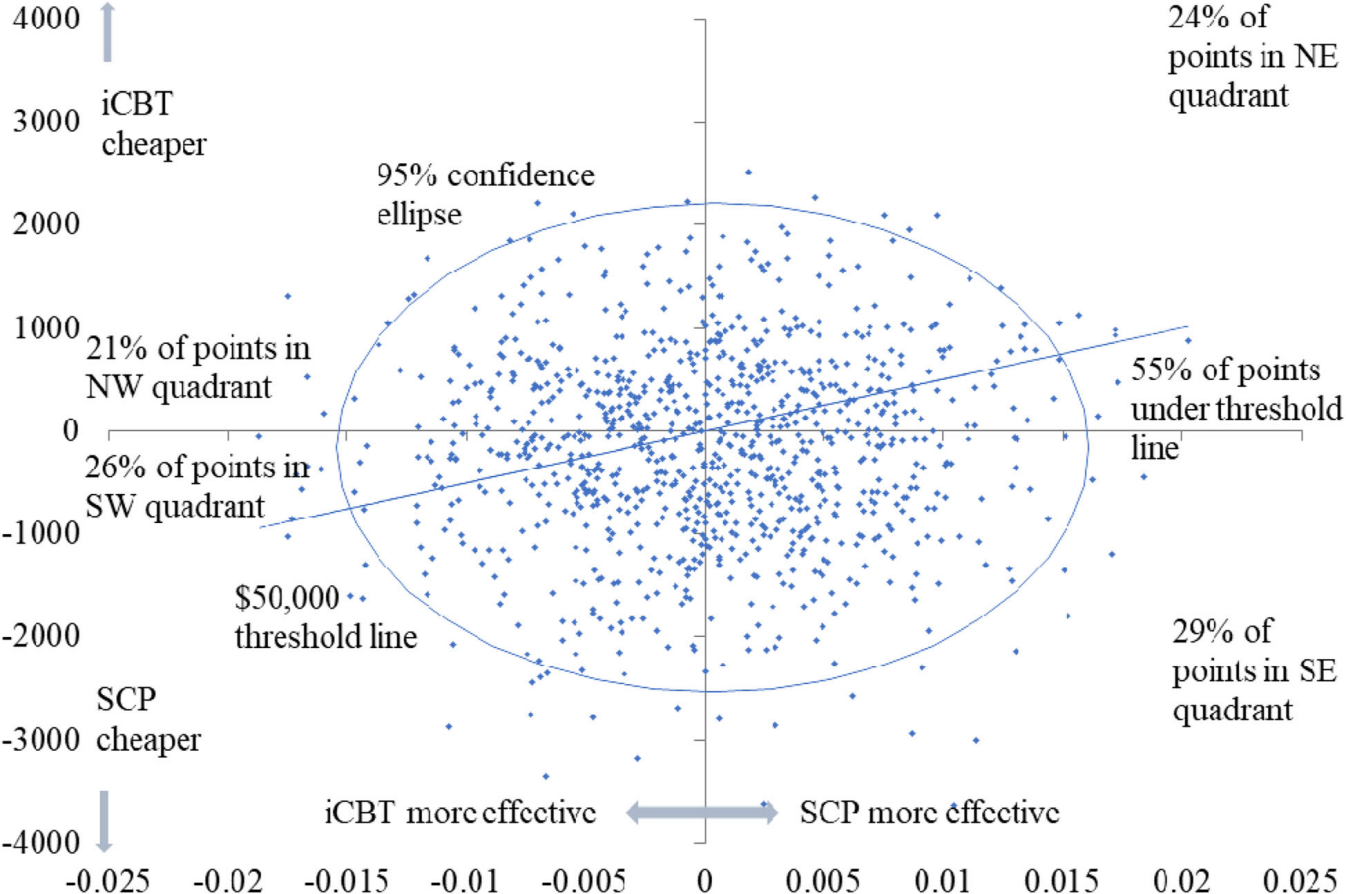
Scatterplot of SCP compared with ICBT for all participants

**Fig. 4:**
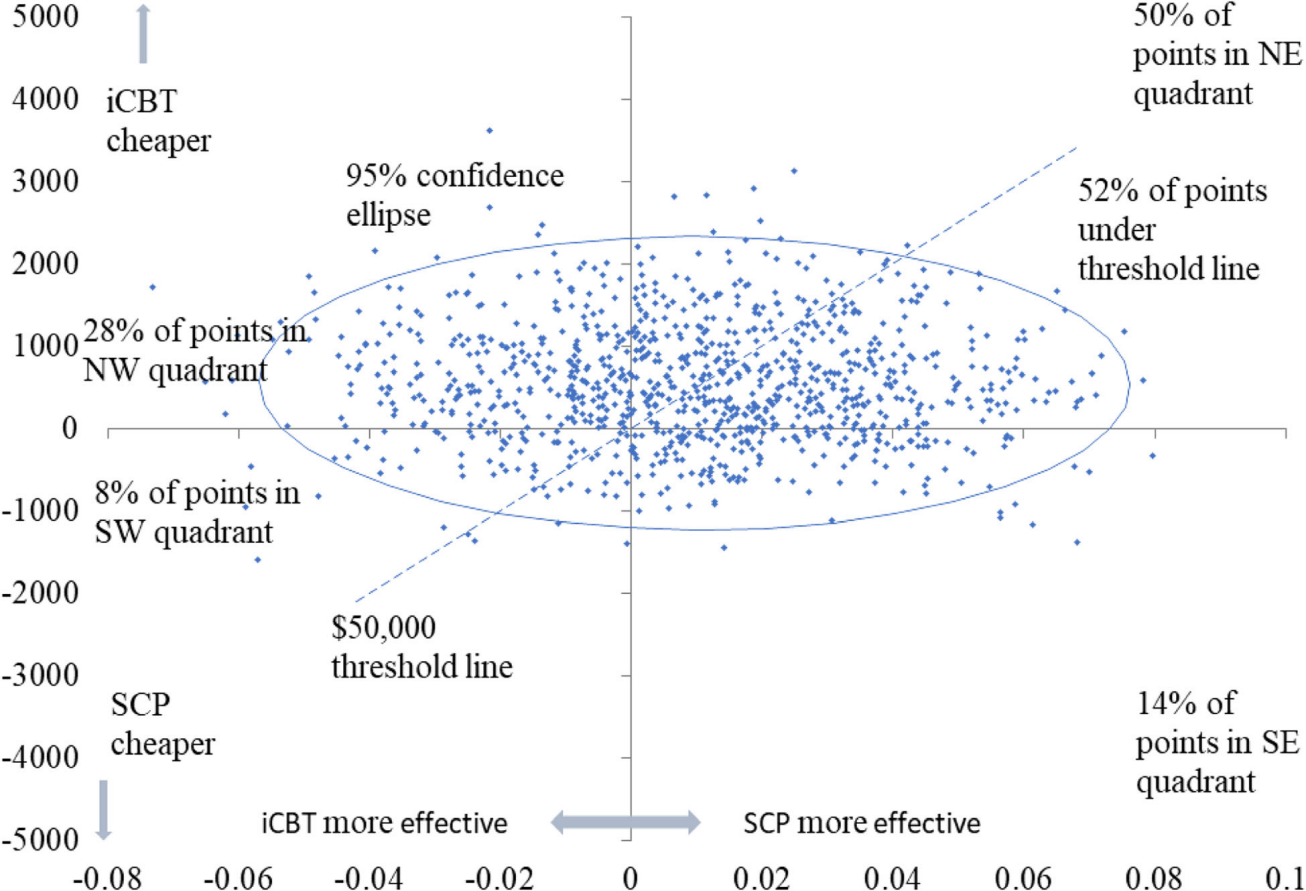
Scatterplot of SCP compared with ICBT for depressed participants

The scatterplot of the simulated costs and QALYs shows pairs of values from the 10,000 simulations on the incremental cost-QALY plane between SCP versus iCBT. Note that scatterplots comparing with SC and TAU were not presented as they were both dominated by iCBT and SCP and were therefore excluded for further consideration. In all participants, there were 55% of scatter points falling under the $50,000 threshold line where SCP was deemed to be cost-effective; in depressed participants, there were 52% of scatter points under the line (Figs. 3 and 4). The scatterplots of TAU compared with SC are available in the Additional file 1.

**Fig. 5:**
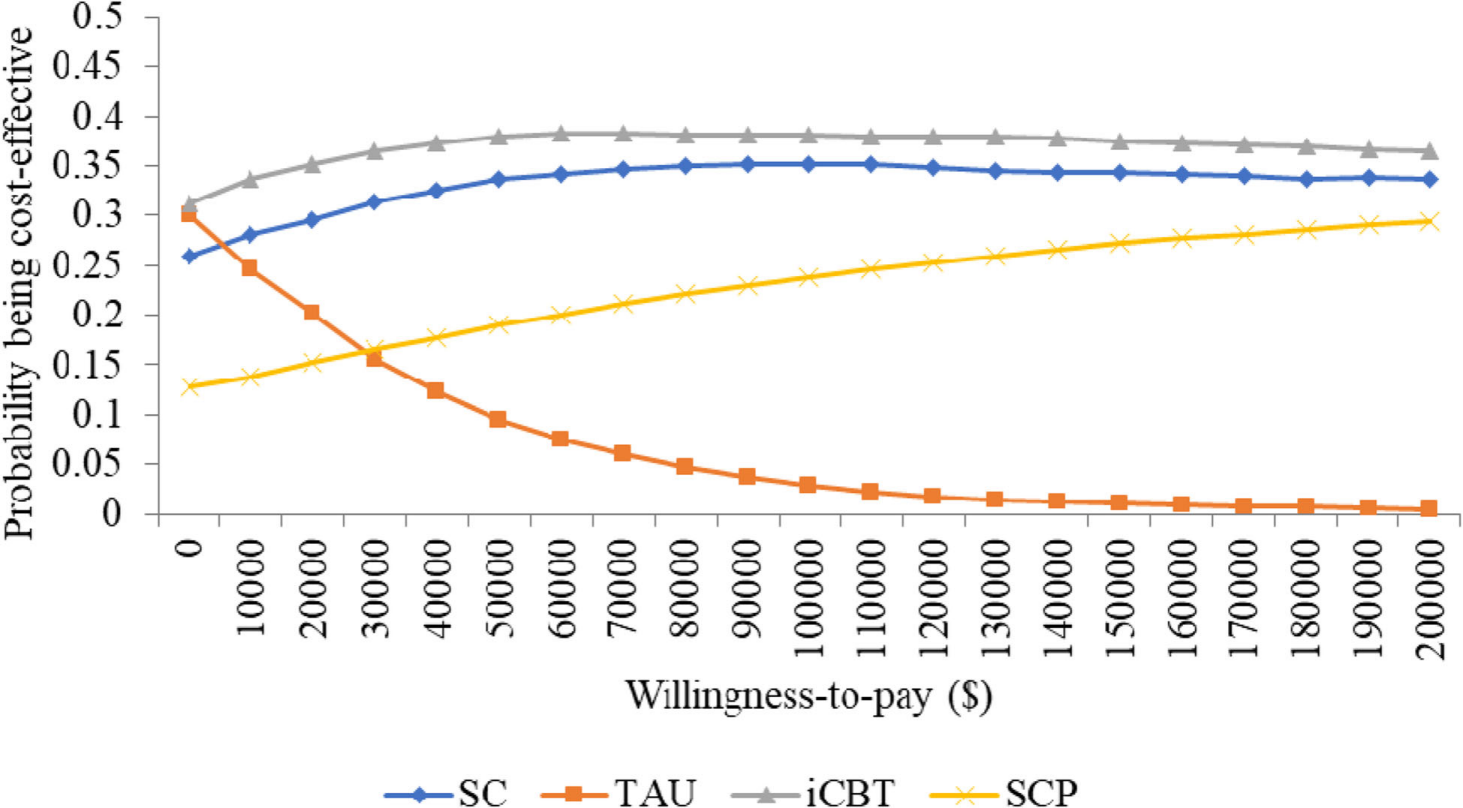
Cost-effectiveness acceptability curves for all participants, sensitivity analysis

**Fig. 6:**
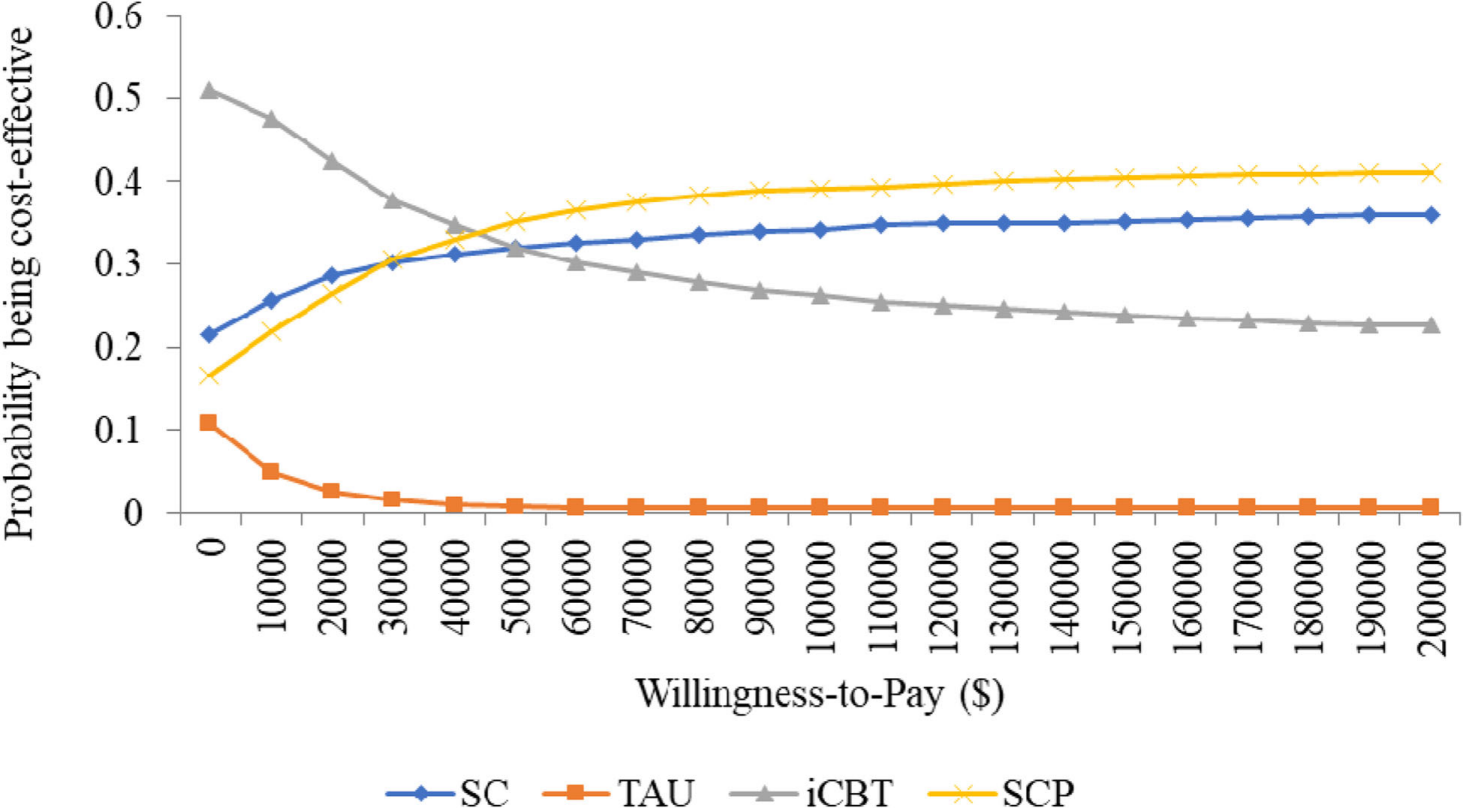
Cost-effectiveness acceptability curves for depressed participants, sensitivity analysis

We expected that the combination of physician, outpatient, and/or inpatient services that a patient received (Table 1) would have a large impact on the cost-effectiveness results, and therefore conducted a sensitivity analysis on a hypothesised scenario that assumed the four intervention arms had identical distribution of patients receiving each combination of medical services (See the rows named “All” in Table 1). In this sensitivity analysis and for all participants, ICBT replaced SCP and became dominating in that it was associated with the highest probability of being cost-effective (Fig. 5). This unsurprising finding was mainly driven by the large increase in the number of theoretical participants receiving physician plus outpatient plus inpatient services in SCP. According to our assumption, this portion in SCP jumped from 9.3% at base-case analysis to 14% in the sensitivity analysis (Table 1). In the subgroup of depressed participants, the results of the sensitivity analysis remained close to the base-case analysis with a slight move leftward of the willingness-to-pay cut-off threshold from approximately $50,000 at base-case analysis to $40,000 per QALY (Fig. 6). This close result between base case analysis and sensitivity analysis was anticipated, given that no substantial changes appeared in this portion (from 12% for ICBT and 11.8% for SCP at base-case analysis to 11.7% at the sensitivity analysis; see Table 1).

To test the assumption that the treatment effect remained unchanged after the 12-week mark for one year, we conducted a sensitivity analysis by assuming (1) the treatment effect gradually reduced to baseline values from 12- to 52-week and (2) the treatment effect immediately reduced to baseline values at 12-week. When giving consideration to the uncertainty in the parameter estimates, we found that the cost-effectiveness results were barely sensitive to the assumptions. The results of the sensitivity analysis are reported in the Additional file 1 in Figures A.3 and A.6.

## Discussion

Our study found that the stepped-care pathway (SCP) for depression was neither superior nor inferior to standard care (SC), treatment as usual (TAU), or treatment as usual plus online-based cognitive behaviour therapy (iCBT) in terms of depression symptom reduction and health-related quality of life (HRQoL) [20]. Interestingly, the present cost-effectiveness analysis suggested SCP is more cost-effective than the other alternatives regardless of how much a decision-maker is willing to pay for a QALY gain. Furthermore, in the subgroup analysis of the depressed participants, SCP is cost-effective against the commonly used willingness-to-pay thresholds of $50,000.

It should be noted that the threshold of $50,000 per QALY is a conservative decision rule. The threshold of $50,000 per QALY has been argued to lack theoretical and empirical justification [45, 46]. Furthermore, it has been suggested that this $50,000 sum has not been adjusted for inflation and changes in increasing healthcare expenditures since its debut in the 1990s [47]. Indeed, others have recommended the use of a willingness-to-pay threshold of $50,000, $100,000, and $200,000 per QALY [45]. If such higher thresholds were used in the current study, then our cost-effectiveness results would be more strongly in favour of SCP.

Interpretation of our results should take into account uncertainty around the cost-effectiveness estimates. Our scatterplots and cost-effectiveness acceptability curves suggested a large degree of uncertainty, making the decision harder when choosing an alternative. While the number of recruited participants was quite high (1400), the final numbers in each arm that had symptoms of depression became relatively small. This lower incidence may contribute considerably to the uncertainty, since a small number is in general associated with larger standard errors and reduces statistical power. We therefore suggest further work with a larger sample size to enhance confidence in the selection between treatment alternatives.

We modelled care costs and considered the combination of physician, outpatient, and inpatient services. Hospital stays are much more expensive than outpatient and physician visits. In our data, hospital stays were approximately eight times the cost of outpatient services and 12 times the cost of physician services, implying hospital stays make up a large percentage of the total cost. Intuitively, if a treatment approach can reduce the likelihood of admittance to a hospital and/or an outpatient visit, then costs will be reduced. We therefore included the distribution of patients receiving each type of care. Our finding that SCP resulted in similar QALY gained but at a financial savings may be driven by a relatively small portion of patients receiving relatively expensive hospital stays. Our sensitivity analysis confirmed this aspect of the study and revealed the cost-effectiveness results were sensitive to the number of patients admitted to hospital.

Among the published cost-effectiveness studies for screening and treatment of depression in primary care ([48–51]), we are not aware of any that simultaneously evaluated the same four intervention alternatives. However, we are aware of studies evaluating the cost-effectiveness of stepped-care pathways (SCP) compared with treatment as usual (TAU), and these studies have reported results consistent with our findings. Thus, Grochtdreis et al. [51] systematically reviewed 19 cost-effectiveness studies that compared stepped-care pathways with treatment as usual for depression in primary care, and found stepped-care pathways were generally more cost-effective. In addition, an economic evaluation in UK primary care [50] demonstrated that improving access to psychological therapies in a stepped approach was cost-effective.

Despite the use of universal screening and an RCT to control some of the biases inherent in such research, we are aware that the present study has limitations. The first is a concern regarding methodology as discussed in our previously published study [20]. Participants were only recruited in two primary care clinics, and while both were trained on the stepped-care pathway, only one of these clinics actively recruited into the stepped-care pathway group. This limited recruitment led to a much smaller sample size in this particular group than the others, and it also had the lowest retention rates for the study. Therefore, clinic differences could, in part, explain some of our cost-effectiveness findings, and we acknowledge this possibility. In addition, dividing the analysis into a subgroup led to the sample size becoming smaller. Secondly, during the course of the original RCT study, one or both clinics could possibly have integrated many of the recommended approaches of the stepped-care pathway into their practices, therefore decreasing the apparent impact of a stepped-care pathway. Thirdly, the effectiveness data was derived from a relatively short study (12-week trial), and we assumed the observed quality of life at 12-week would be maintained until one year. This assumption may be debatable. We tested the assumption by examining two other scenarios in which the treatment effect was assumed to vanish immediately at 12-week and to reduce gradually to baseline values at 52-week. The analysis found little change in the cost-effectiveness results.

The fourth possible limitation is that implementation costs were not included in the economic analysis. There would be set-up costs for the stepped-care pathway, and excluding these costs would underestimate the total costs. However, since these additional costs would occur only during the program implementation period, their effect on the intervention’s overall cost-effectiveness is likely limited over the longer term. Finally, people with major depressive disorder experience higher rates of unemployment and other disabilities than their peers [6]. This aspect may have an impact on societal costs due to productivity losses and increasing use of care givers and/or social workers. However, because the analysis specifically focuses on the health care system, costs outside the scope were not considered. Gender difference in both health care usage and depression symptoms is also worth keeping in mind when interpreting our results. Female participants account for around 70% of total participants in our analysis. This proportion shows a similar trend suggested in the previous literature in which women had much higher rates of depressive disorder compared to men [52, 53]. Our analysis did not split the resource use and costs by gender, as it was predicated on universal screening at a family practice and so is presumed to represent patients using the system. In addition, our results are for patients who go to an appointment at a primary care physician. So overall costs would be altered by the impact of people who do not see a doctor at all.

## Conclusion

The present economic analysis finds that even where there are no clinically significant differences in health outcome, economic savings may arise from implementing the stepped-care model. As with previous studies, we found that individuals who had depression incurred greater health care costs than those who were not depressed. However, somewhat counter-intuitively, our results suggest that a more comprehensive stepped-care pathway may lead to significant savings in overall health care costs versus treatment as usual for the entire population, not only those who screened as depressed at baseline. These findings, if validated in other studies, could have major economic implications for health care systems. As stepped-care pathways have been increasingly adopted by clinicians and policy makers [15, 16, 22], the economic impact of this treatment approach typically causes concern [19, 20]. Our results, if supported by future research, would help address these issues and possibly assist health care planners in making more informed choices. While more work is required to identify the most clinically effective versions of a stepped-care pathway, our findings suggest that the care pathway may have substantial potential to improve health care system value in terms of a lower incremental cost-effectiveness ratio compared with treatment as usual and online-based cognitive behaviour therapy.

## Additional file

Additional file 1: **Table S1.** Number of participants in the four treatment groups separated into depressed and non-depressed subgroups. (DOCX 207 kb)

## Data Availability

We are part of the research team who has access to datasets of the study for publications. Governed by privacy legislation and agreements between the research team and provider agencies, the primary population health and economic data are not part of datasets available for other analyses.

## Abbreviations

CBT: Cognitive Behavioural Therapy
CI: Confidence Interval
CPI: Consumer Price Index
EQ-5D-5 L: Euroqol-5-Dimension with five level scale
GLM: Generalized Linear Model
HRQoL: Health-Related Quality of Life
iCBT: Online Cognitive Behavioural Therapy
ICER: Incremental Cost-Effectiveness Ratio
IQR: Interquartile Range
ISPOR: International Society for Pharmacoeconomics and Outcomes Research
ITT: Intention-To-Treat
OLS: Ordinary Least Squares
PHQ-9: Patient Health Questionnaire – 9-Item
QALY: Quality-Adjusted Life-Year
RCT: Randomized, Controlled Trial
s.d.: Standard Deviation
SC: Standard Care
SCP: Stepped-Care Pathway
TAU: Treatment-as-Usual
