# Supplementary material for: Cost-effectiveness analysis of a randomized study of depression treatment options in primary care suggests stepped-care treatment may have economic benefits
Source: BMC Psychiatry. 2019 Aug 5;19:240. doi: 10.1186/s12888-019-2223-3 (PMC6683422; doi:10.1186/s12888-019-2223-3)
Supplement: Supplementary file 1 — Table S1. Number of participants in the four treatment groups separated into depressed and non-depressed subgroups. (DOCX 207 kb) [file 12888_2019_2223_MOESM1_ESM.docx]

Additional file 1

### Table S1. Number of participants in the four treatment groups separated into depressed and non-depressed subgroups

| **Study Arm** | **Baseline** | | | **12-week post randomization** | | | **Difference** | | |
| --- | --- | --- | --- | --- | --- | --- | --- | --- | --- |
|  | **non depressed** | **depressed** | **overall** | **non depressed** | **depressed** | **overall** | **non depressed** | **depressed** | **overall** |
| SC | 356 | 56 | 412 | 225 | 40 | 265 | 131 | 16 | 147 |
| TAU | 331 | 66 | 397 | 223 | 44 | 267 | 108 | 22 | 130 |
| iCBT | 365 | 50 | 415 | 216 | 29 | 245 | 149 | 21 | 170 |
| SCP | 149 | 34 | 183 | 57 | 14 | 71 | 92 | 20 | 112 |
| Total | 1201 | 206 | 1407 | 721 | 127 | 848 | 480 | 79 | 559 |

### Table A.2 Physician cost per patient by period, treatment groups and patient subgroup

| **Overall** | | | | | | | | |
| --- | --- | --- | --- | --- | --- | --- | --- | --- |
| **Period** | **arm** | **mean** | **sd** | **min** | **max** | **N** | **95% CI** | |
| 12 weeks before | SC | $235.26 | $49.14 | $150.79 | $401.18 | 255 | 229.20 | 241.32 |
|  | TAU | $243.43 | $24.37 | $184.72 | $331.48 | 256 | 240.43 | 246.43 |
|  | iCBT | $238.48 | $43.85 | $56.26 | $345.45 | 251 | 233.03 | 243.94 |
|  | SCP | $224.47 | $54.56 | $113.61 | $356.96 | 119 | 214.56 | 234.37 |
| 12 weeks after | SC | $250.83 | $51.34 | $89.48 | $468.91 | 343 | 245.37 | 256.28 |
|  | TAU | $317.83 | $52.64 | $237.16 | $510.08 | 328 | 312.11 | 323.55 |
|  | iCBT | $256.96 | $43.78 | $152.42 | $357.06 | 342 | 252.31 | 261.62 |
|  | SCP | $265.62 | $62.83 | $127.21 | $488.45 | 162 | 255.87 | 275.37 |
| 1 year | SC | $546.13 | $111.72 | $212.50 | $838.57 | 337 | 534.16 | 558.10 |
|  | TAU | $560.96 | $124.76 | $225.94 | 1,024.29 | 321 | 547.26 | 574.66 |
|  | iCBT | $522.72 | $50.03 | $441.39 | $767.61 | 335 | 517.34 | 528.10 |
|  | SCP | $414.98 | $60.18 | $265.05 | $515.13 | 128 | 404.45 | 425.50 |
| **Non-depressed** | | | | | | | | |
| 12 weeks before | SC | $220.69 | $33.83 | $150.79 | $298.17 | 215 | 216.14 | 225.23 |
|  | TAU | $235.87 | $18.15 | $184.72 | $276.13 | 210 | 233.40 | 238.34 |
|  | iCBT | $233.15 | $35.55 | $56.26 | $274.31 | 214 | 228.36 | 237.94 |
|  | SCP | $215.85 | $51.10 | $113.61 | $321.75 | 95 | 205.44 | 226.26 |
| 12 weeks after | SC | $238.79 | $36.15 | $89.48 | $336.39 | 298 | 234.67 | 242.91 |
|  | TAU | $298.58 | $29.93 | $237.16 | $374.30 | 271 | 295.00 | 302.16 |
|  | iCBT | $253.29 | $42.53 | $152.42 | $357.06 | 295 | 248.41 | 258.16 |
|  | SCP | $245.46 | $39.98 | $127.21 | $354.36 | 133 | 238.60 | 252.31 |
| 1 year | SC | $536.47 | $101.85 | $212.50 | $780.65 | 293 | 524.76 | 548.19 |
|  | TAU | $528.00 | $94.16 | $225.94 | $745.84 | 272 | 516.76 | 539.24 |
|  | iCBT | $513.63 | $42.73 | $441.39 | $744.24 | 293 | 508.72 | 518.54 |
|  | SCP | $413.78 | $58.80 | $270.65 | $515.13 | 106 | 402.46 | 425.10 |
| **Depressed** | | | | | | | | |
| 12 weeks before | SC | $313.58 | $44.56 | $239.23 | $401.18 | 40 | 299.33 | 327.83 |
|  | TAU | $277.95 | $18.74 | $222.10 | $331.48 | 46 | 272.39 | 283.52 |
|  | iCBT | $269.35 | $68.72 | $110.19 | $345.45 | 37 | 246.44 | 292.27 |
|  | SCP | $258.60 | $55.49 | $122.01 | $356.96 | 24 | 235.17 | 282.03 |
| 12 weeks after | SC | $330.52 | $64.71 | $151.40 | $468.91 | 45 | 311.08 | 349.96 |
|  | TAU | $409.33 | $39.34 | $335.06 | $510.08 | 57 | 398.89 | 419.76 |
|  | iCBT | $280.04 | $44.90 | $159.15 | $347.54 | 47 | 266.86 | 293.22 |
|  | SCP | $358.12 | $66.13 | $204.63 | $488.45 | 29 | 332.96 | 383.27 |
| 1 year | SC | $610.45 | $149.07 | $250.98 | $838.57 | 44 | 565.12 | 655.77 |
|  | TAU | $743.94 | $115.64 | $428.66 |  | 49 | 710.73 | 777.16 |
|  | iCBT | $586.14 | $51.69 | $505.96 | $767.61 | 42 | 570.03 | 602.24 |
|  | SCP | $420.74 | $67.67 | $265.05 | $505.78 | 22 | 390.74 | 450.74 |

### Table A.3 Outpatient cost per patient by period, treatment groups and patient subgroup

| **Overall** | | | | | | | | |
| --- | --- | --- | --- | --- | --- | --- | --- | --- |
| **Period** | **arm** | **mean** | **sd** | **min** | **max** | **N** | **95% CI** | |
| 12 weeks before | SC | $728.24 | $38.00 | $668.60 | $942.17 | 81 | 719.83 | 736.64 |
|  | TAU | $663.70 | $136.83 | $250.27 | $1,050.01 | 85 | 634.18 | 693.21 |
|  | iCBT | $635.80 | $108.76 | $141.16 | $736.12 | 99 | 614.11 | 657.49 |
|  | SCP | $616.28 | $218.50 | $146.86 | $1,057.39 | 37 | 543.43 | 689.13 |
| 12 weeks after | SC | $670.10 | $112.11 | $525.37 | $1,032.68 | 94 | 647.13 | 693.06 |
|  | TAU | $584.66 | $71.22 | $386.81 | $837.37 | 102 | 570.67 | 598.65 |
|  | iCBT | $551.77 | $47.71 | $479.95 | $673.63 | 96 | 542.10 | 561.44 |
|  | SCP | $623.28 | $169.99 | $355.02 | $1,054.88 | 40 | 568.91 | 677.64 |
| 1 year | SC | $935.88 | $115.87 | $723.64 | $1,236.24 | 152 | 917.31 | 954.45 |
|  | TAU | $891.31 | $188.61 | $517.05 | $1,289.29 | 158 | 861.67 | 920.95 |
|  | iCBT | $869.71 | $111.91 | $490.87 | $1,195.65 | 149 | 851.59 | 887.82 |
|  | SCP | $1,069.49 | $345.10 | $103.30 | $1,938.31 | 73 | 988.97 | 1,150.01 |
| **Non-depressed** | | | | | | | | |
| 12 weeks before | SC | $730.57 | $11.61 | $707.36 | $750.49 | 64 | 727.67 | 733.47 |
|  | TAU | $622.39 | $90.45 | $250.27 | $774.17 | 69 | 600.66 | 644.12 |
|  | iCBT | $659.49 | $83.39 | $243.06 | $736.12 | 87 | 641.72 | 677.26 |
|  | SCP | $503.21 | $142.82 | $146.86 | $773.22 | 25 | 444.26 | 562.16 |
| 12 weeks after | SC | $624.74 | $63.05 | $525.37 | $765.24 | 73 | 610.03 | 639.45 |
|  | TAU | $558.73 | $38.72 | $386.81 | $635.07 | 82 | 550.23 | 567.24 |
|  | iCBT | $547.71 | $45.95 | $479.95 | $661.24 | 81 | 537.55 | 557.87 |
|  | SCP | $539.43 | $98.09 | $355.02 | $751.53 | 29 | 502.12 | 576.74 |
| 1 year | SC | $916.05 | $105.47 | $723.64 | $1,139.64 | 129 | 897.68 | 934.43 |
|  | TAU | $862.05 | $184.17 | $517.05 | $1,280.65 | 127 | 829.71 | 894.39 |
|  | iCBT | $846.58 | $88.62 | $490.87 | $1,029.14 | 132 | 831.32 | 861.84 |
|  | SCP | $934.95 | $258.67 | $103.30 | $1,371.78 | 53 | 863.65 | 1,006.25 |
| **Depressed** | | | | | | | | |
| 12 weeks before | SC | $719.43 | $81.14 | $668.60 | $942.17 | 17 | 677.71 | 761.15 |
|  | TAU | $841.83 | $161.44 | $523.06 | $1,050.01 | 16 | 755.80 | 927.85 |
|  | iCBT | $464.02 | $119.93 | $141.16 | $556.03 | 12 | 387.82 | 540.22 |
|  | SCP | $851.85 | $148.83 | $580.02 | $1,057.39 | 12 | 757.28 | 946.41 |
| 12 weeks after | SC | $827.76 | $102.57 | $592.66 | $1,032.68 | 21 | 781.07 | 874.45 |
|  | TAU | $690.97 | $76.00 | $527.16 | $837.37 | 20 | 655.40 | 726.54 |
|  | iCBT | $573.69 | $52.62 | $488.19 | $673.63 | 15 | 544.55 | 602.83 |
|  | SCP | $844.33 | $107.79 | $656.33 | $1,054.88 | 11 | 771.92 | 916.74 |
| 1 year | SC | $1,047.06 | $110.47 | $821.13 | $1,236.24 | 23 | 999.29 | 1,094.84 |
|  | TAU | $1,011.20 | $158.85 | $692.20 | $1,289.29 | 31 | 952.94 | 1,069.47 |
|  | iCBT | $1,049.29 | $113.42 | $774.84 | $1,195.65 | 17 | 990.97 | 1,107.60 |
|  | SCP | $1,426.03 | $289.61 | $641.22 | $1,938.31 | 20 | 1,290.49 | 1,561.57 |

### Table A.4 Inpatient cost per patient by period, treatment groups and patient subgroup

| **Overall** | | | | | | | | |
| --- | --- | --- | --- | --- | --- | --- | --- | --- |
| **Period** | **arm** | **mean** | **sd** | **min** | **max** | **N** | **Add 95% CI** | |
| 12 weeks before | SC | $8,439.91 | $4,553.39 | $4,016.72 | $16,933.26 | 17 | 6,275.37 | 10,604.45 |
|  | TAU | $8,851.42 | $3,048.50 | $4,832.39 | $13,469.28 | 12 | 7,126.57 | 10,576.28 |
|  | iCBT | $8,678.92 | $3,754.46 | $3,337.87 | $15,504.53 | 28 | 7,288.24 | 10,069.59 |
|  | SCP | $10,442.75 | $3,021.12 | $6,259.81 | $13,400.87 | 4 | 7,482.06 | 13,403.44 |
| 12 weeks after | SC | $6,251.12 | $2,456.10 | $3,335.84 | $11,890.34 | 23 | 5,247.34 | 7,254.90 |
|  | TAU | $5,727.25 | $2,423.70 | $2,768.02 | $13,184.56 | 31 | 4,874.04 | 6,580.46 |
|  | iCBT | $5,745.92 | $2,409.87 | $3,457.20 | $13,549.52 | 26 | 4,819.60 | 6,672.25 |
|  | SCP | $8,862.27 | $2,820.16 | $5,646.98 | $10,916.81 | 3 | 5,670.96 | 12,053.57 |
| 1 year | SC | $9,061.01 | $4,505.12 | $3,739.25 | $18,033.13 | 34 | 7,546.67 | 10,575.35 |
|  | TAU | $9,059.03 | $4,661.99 | $2,968.07 | $19,157.38 | 43 | 7,665.58 | 10,452.49 |
|  | iCBT | $10,084.45 | $4,285.85 | $4,027.92 | $17,551.22 | 41 | 8,772.55 | 11,396.35 |
|  | SCP | $9,551.24 | $4,156.60 | $4,317.18 | $16,330.94 | 15 | 7,447.71 | 11,654.77 |
| **Non-depressed** | | | | | | | | |
| 12 weeks before | SC | $8,439.91 | $4,553.39 | $4,016.72 | $16,933.26 | 17 | 6,275.37 | 10,604.45 |
|  | TAU | $8,397.47 | $3,083.84 | $4,832.39 | $11,835.32 | 7 | 6,112.93 | 10,682.01 |
|  | iCBT | $8,574.21 | $3,886.10 | $3,337.87 | $15,504.53 | 25 | 7,050.86 | 10,097.57 |
|  | SCP | $8,876.54 | $3,700.61 | $6,259.81 | $11,493.27 | 2 | 3,747.76 | 14,005.33 |
| 12 weeks after | SC | $5,851.03 | $2,199.99 | $3,335.84 | $11,240.14 | 20 | 4,886.84 | 6,815.22 |
|  | TAU | $5,356.79 | $2,021.69 | $2,768.02 | $13,184.56 | 29 | 4,620.97 | 6,092.61 |
|  | iCBT | $5,697.97 | $2,446.87 | $3,457.20 | $13,549.52 | 25 | 4,738.80 | 6,657.15 |
|  | SCP | $7,834.99 | $3,094.32 | $5,646.98 | $10,023.01 | 2 | 3,546.48 | 12,123.51 |
| 1 year | SC | $9,032.72 | $4,578.03 | $3,739.25 | $18,033.13 | 30 | 7,394.50 | 10,670.95 |
|  | TAU | $8,739.61 | $4,789.11 | $2,968.07 | $19,157.38 | 37 | 7,196.46 | 10,282.77 |
|  | iCBT | $10,278.98 | $4,384.15 | $4,027.92 | $17,551.22 | 37 | 8,866.31 | 11,691.65 |
|  | SCP | $9,930.62 | $4,589.78 | $4,317.18 | $16,330.94 | 12 | 7,333.71 | 12,527.53 |
| **Depressed** | | | | | | | | |
| 12 weeks before | SC |  |  |  |  | 0 |  |  |
|  | TAU | $9,486.96 | $3,228.95 | $5,720.40 | $13,469.28 | 5 | 6,656.66 | 12,317.26 |
|  | iCBT | $9,551.42 | $2,791.92 | $6,336.09 | $11,361.69 | 3 | 6,392.07 | 12,710.78 |
|  | SCP | $12,008.96 | $1,968.46 | $10,617.05 | $13,400.87 | 2 | 9,280.82 | 14,737.10 |
| 12 weeks after | SC | $8,918.36 | $2,846.90 | $6,215.68 | $11,890.34 | 3 | 5,696.79 | 12,139.93 |
|  | TAU | $11,098.85 | $315.07 | $10,876.06 | $11,321.64 | 2 | 10,662.19 | 11,535.51 |
|  | iCBT | $6,944.72 | . | $6,944.72 | $6,944.72 | 1 | . | . |
|  | SCP | $10,916.81 | . | $10,916.81 | $10,916.81 | 1 | . | . |
| 1 year | SC | $9,273.17 | $4,537.84 | $5,089.56 | $14,372.89 | 4 | 4,826.09 | 13,720.25 |
|  | TAU | $11,028.78 | $3,466.97 | $5,024.55 | $15,311.18 | 6 | 8,254.63 | 13,802.93 |
|  | iCBT | $8,285.04 | $3,079.02 | $5,281.61 | $12,444.65 | 4 | 5,267.61 | 11,302.48 |
|  | SCP | $8,033.70 | $871.71 | $7,530.12 | $9,040.27 | 3 | 7,047.27 | 9,020.14 |

Figure A.1 Scatterplot of TAU compared with SC for all participants

Figure A.2 Scatterplot of TAU compared with SC for depressed participants

Figure A.3 Cost-effectiveness acceptability curves for all participants, assuming treatment effect reduced gradually to baseline values from 12- to 52-week

Figure A.4 Cost-effectiveness acceptability curves for all participants, assuming treatment effect reduced immediately to baseline values at 12-week

Figure A.5 Cost-effectiveness acceptability curves for depressed participants, assuming treatment effect reduced gradually to baseline values from 12- to 52-week

Figure A.6 Cost-effectiveness acceptability curves for depressed participants, assuming treatment effect reduced immediately to baseline values at 12-week
